# Co-creation and interprofessional collaborative practice for the local management of osteoporosis: a qualitative study in primary healthcare

**DOI:** 10.1093/fampra/cmag045

**Published:** 2026-06-23

**Authors:** Jenny Nilsson, Helena Salminen, Eva Toth-Pal, Hans Ranch Lundin

**Affiliations:** Division of Family Medicine and Primary Care, Department of Neurobiology, Care Sciences and Society, Karolinska Institutet, 171 77 Stockholm, Sweden; Division of Family Medicine and Primary Care, Department of Neurobiology, Care Sciences and Society, Karolinska Institutet, 171 77 Stockholm, Sweden; Academic Primary Health Care Centre, Box 45436, 104 31 Stockholm, Sweden; Division of Family Medicine and Primary Care, Department of Neurobiology, Care Sciences and Society, Karolinska Institutet, 171 77 Stockholm, Sweden; Division of Family Medicine and Primary Care, Department of Neurobiology, Care Sciences and Society, Karolinska Institutet, 171 77 Stockholm, Sweden; Academic Primary Health Care Centre, Box 45436, 104 31 Stockholm, Sweden

**Keywords:** interprofessional relations, patient care team, community-based participatory research, primary healthcare, osteoporosis, osteoporotic fractures

## Abstract

**Background:**

Interprofessional collaborative practice (ICP) benefits patients with complex conditions such as osteoporosis that require care from multiple professions. ICP is not consistently applied in osteoporosis management in primary healthcare today.

**Objectives:**

To explore how an ICP intervention promoting co-creation of osteoporosis management was conducted and how it was experienced by nurses and physicians in primary healthcare.

**Methods:**

The intervention part was guided by participatory health research approach. In total, 12 nurses and physicians from five primary healthcare centres participated in three workshops aimed at supporting co-creation of local osteoporosis management. The evaluation part consisted of group and individual interviews and surveys capturing self-reported experiences. Data were analysed using qualitative content analysis with inductive approach.

**Results:**

Participants reviewed local osteoporosis management and co-created work procedures tailored to their settings. The analysis generated three categories with seven subcategories. The categories were innovative learning processes, perceived effects, and factors influencing changes in work procedures. Innovative learning processes described shared learning, reflection, and adaptation in developing work procedures. Perceived effects included expected benefits for patients, changes in professional roles, and increased interprofessional collaboration. Participants described a strengthened role for nurses in osteoporosis management. Factors influencing changes in work procedures included organizational, professional, and resource-related conditions. The survey findings supported those from the interviews.

**Conclusion:**

A participatory approach facilitated collaborative revision of osteoporosis management by leveraging local knowledge and promoting interprofessional learning. Participatory health research appears useful for developing ICP in managing complex conditions such as osteoporosis in primary healthcare.

Key messages

- Co-creation and co-learning may improve management of complex conditions.
- Participatory approaches help adapt care and foster team collaboration.
- Well-facilitated workshops allowed teams to share experiences and find solutions.
- Wider use may need organizational support for feasibility and sustainability.

## Background

Interprofessional collaborative practice (ICP) benefits both patients and healthcare professionals by decreasing hospital admissions, shortening stays, and reducing complications [1]. Moreover, ICP can improve health outcomes and patient satisfaction [1]. The practice can be described as a form of teamwork in which various healthcare professions and the patient collaborate to achieve a common goal [1, 2]. In primary healthcare, ICP typically brings together physicians and nurses who share responsibility for managing chronic conditions. Collaboration is a routine part of care, but its structure varies between primary healthcare centres (PHCCs) and depends on organizational conditions, local work procedures, and professional roles [3–7]. The organization and application of collaboration in osteoporosis care also differ between PHCCs. These differences highlight the need for approaches that enable healthcare professionals to collaboratively adapt work procedures to their local context through co-creation [8–10]. There are, however, certain prerequisites if ICP is to succeed: team members must be motivated and have organizational support, collaborative competence, and a culture that promotes collaboration [1, 6, 11]. Developing these competences often involves self-directed learning, where professionals take responsibility for their learning needs, and experiential learning, emphasizing practical experience and reflection [12, 13].

Osteoporosis management is a good example of an approach requiring multiprofessional care. In primary healthcare, osteoporosis management is often complicated by multimorbidity, high patient age, and competing demands from comorbid conditions [5, 14–16]. The patient population is heterogeneous, which can complicate identification and management [15, 16]. Osteoporosis is often asymptomatic until fracture occurs, adding to these challenges [15–18].

Osteoporosis is a common condition that causes fragility fractures. It is often underdiagnosed and undertreated, and there is a significant treatment gap [15–17, 19]. Treatment has been shown to be effective and to reduce the risk of fractures [20]. The most common fracture sites are wrists, hips, and vertebrae [21–23], causing suffering and high societal costs [17, 19, 24]. Osteoporosis management is a comprehensive approach to reducing the fracture risk, primarily through secondary prevention [17, 21–23, 25]. This includes pharmacotherapy and non-pharmacological interventions, such as fall prevention, physical activity, nutritional support, and patient education. ICP can facilitate osteoporosis management by supporting coordination between professionals involved in diagnosis, treatment, follow-up, and patient education. A clearer division of roles and shared work procedures promote more consistent and coordinated care [1, 6, 7, 25].

Participatory health research is an approach in which researchers and stakeholders are co-creators in the process of generating new knowledge [26, 27]. It is particularly suitable for creating and studying ICP within the healthcare setting, as it supports collaboration and the development of practices adapted to local contexts [26, 27]. This approach emphasizes collaboration, mutual learning, and reflection and brings together practical experience with research [26, 27]. Osteoporosis management is an area in which more participatory health research is needed to understand why the treatment gap persists despite the introduction of national guidelines [14, 16, 17].

Given the documented treatment gap, we assumed that engaging nurses and physicians in interprofessional collaboration to co-create and improve local work procedures would foster conditions supporting ICP [15–17]. This qualitative study aimed to explore the experiences of nurses and physicians of an intervention to enable them to co-create local work procedures for osteoporosis management in primary healthcare, and their perceptions of its impact on their own PHCC. Additionally, we studied how the resulting changes were integrated into routine practice.

## Materials and methods

### Design

This pilot study was guided by participatory health research approach, based on the participatory framework described by Wright *et al*. [27]. In line with Wright *et al*. [27], participatory health research is understood as a research framework that emphasises co-creation of knowledge and active participation throughout the research process. The research questions were formulated by the researchers, while participants played an active role in co-creating local work procedures for osteoporosis management and integrating them into their practice. This approach reflects the levels of co-operation and co-learning described by Wright *et al*. [27].

The study was grounded in a social constructivist paradigm, assuming that understanding of interprofessional practice develops through interaction between participants [28]. This epistemological perspective aligns with participatory health research, as both emphasize that knowledge is co-constructed through social interaction [27]. The participatory approach guided the intervention, and the analysis focused on participants’ experiences of the co-creation process as expressed in interviews and surveys.

### Researcher characteristics and reflexivity

The first author (J.N.) is a doctoral student and a physiotherapist with clinical experience in Swedish primary healthcare. She has experience of quantitative research unrelated to osteoporosis and interprofessional collaboration. The co-authors (H.S., E.T.P., and H.L.) are family physicians and senior researchers with experience in both quantitative and qualitative research and studies of osteoporosis and interprofessional education. We acknowledged these backgrounds and engaged in reflexive discussions throughout the study to surface assumptions and maintain transparency. The first author's role as both observer and interviewer was considered during analysis, and the authors engaged in reflexive discussions to critically examine interpretations.

### Context

The study was conducted in primary healthcare in Region Stockholm, Sweden. Primary healthcare is the first line of care, managing a wide range of conditions from minor illnesses to complex and often chronic diseases [5, 29]. Patients are registered at a specific PHCC, which coordinates their care [29]. Responsibilities in primary healthcare have expanded over time, and emphasis on team-based care and shared management of chronic conditions has increased [5, 7, 30]. Patient care in primary healthcare often involves several professions, with physicians and nurses being the main groups holding independent responsibility. The organization of care and use of ICP vary between PHCCs. ICP is an important component in effective osteoporosis management. Collaborative practices across PHCCs differ in terms of structure and extent. Clear roles and care processes are not always in place in clinical practice.

### Participants

Emails were sent to clinic managers at 218 PHCCs, inviting them to participate in the study. Information was also shared at staff meetings. Each participating PHCC formed an interprofessional team of nurses and physicians (IP team). A total of five PHCCs agreed to participate in the pilot study. The interprofessional teams consisted of three teams with one nurse and one physician, one team with two nurses and one physician, and one team with one nurse and two physicians. In total, 12 participants were included (see Table 1).

**Table 1:** Descriptive characteristics of participants.

	All participants (*n* = 12)	Nurses (*n* = 6)	Physicians (*n* = 6)
Female	10	4	6
Male	2	2	0
Age (median, range)	39.5 (35–61)	36.5 (35–60)	43 (37–61)

### Intervention

The intervention consisted of an initial online review of osteoporosis management and workshops. To ensure a common understanding and terminology within IP teams, all participants were encouraged to take part in the short online review of osteoporosis, falls, and fragility fractures. Participants engaged with the material independently and used it as a basis for subsequent workshop discussions. The roles of all healthcare professions were highlighted, not just physicians and nurses. Created by the researchers, the review consisted of short screencasts with audio narration and documents to read, including content based on national osteoporosis guidelines. It was held asynchronously on an online teaching and learning platform.

Online workshops, facilitated by a moderator (H.L.), aimed to support co-creation and co-learning within each IP team and to improve local osteoporosis management procedures. An observer (J.N.) took field notes, helped with timekeeping, and recorded the workshops (audio and video). Participants were given discussion points to encourage dialogue, and the moderator facilitated discussions. The workshops involved facilitated discussions, reflection on current practice, and collaborative planning and testing of changes to work procedures. The moderator introduced discussion topics to guide the discussions and let participants shape the content and focus of each workshop. This approach supported collaborative reflection and learning. Participants also presented and discussed work carried out between workshops and shared experiences within and across teams. Each workshop lasted about 90 minutes.

During the first workshop, participants described existing osteoporosis management procedures and explored strategies to identify more patients at risk of fragility fractures. They also discussed actions available when a high-risk patient was identified but preventive treatment was inadequate. A final discussion focused on collaboration between physicians and nurses. In the 4-week period between the first and second workshop, IP teams continued their discussions and revised work procedures within the IP team, as well as engaging other staff at their PHCC.

In the second workshop, participants followed up on how discussions and revisions had taken shape at their PHCC during the preceding 4 weeks. They discussed changes to existing procedures and how to introduce or trial these changes. They also considered factors that supported or undermined the process. In the next 4-week period, participants were encouraged to present their proposed changes to staff at their PHCC and to begin putting the revised procedures into practice.

The third workshop began by following up the IP teams’ progress in revising procedures. Participants explained how they had presented proposed changes to other staff at their PHCC and reflected on their reactions and level of interest. Plans for ongoing activities to improve the identification and care of high-risk patients at their PHCC were also discussed.

### Data collection

**Figure 1:**
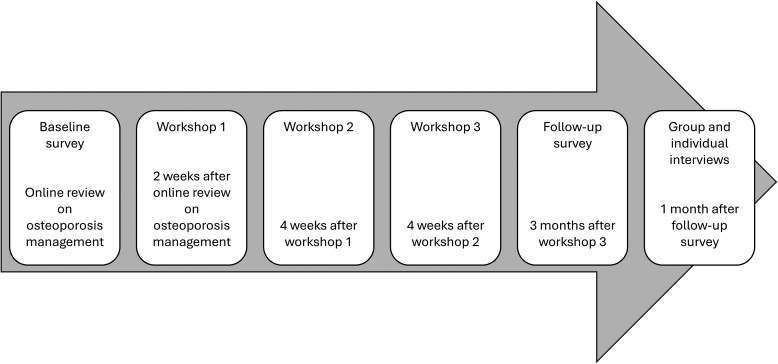
Flow chart of intervention and data collection. The chart illustrates the timeline of the intervention and data collection process. It includes the baseline survey, three workshops, follow-up survey, and subsequent interviews, with intervals between each step.

Data were collected through surveys and interviews, providing data from multiple sources (Fig. 1 provides an overview) [31]. Participants completed a web-based survey before the study and another 3 months after the third workshop (see Supplementary S1). The surveys developed for this study covered background information, the organization of osteoporosis management at PHCCs and perceptions of fragility fracture prevention. Responses were graded on a four-point Likert scale (1 = Not at all, 2 = To a small extent, 3 = To a considerable extent, 4 = To a great extent). The second survey repeated the first and added items that explored participants’ experiences of revising work procedures and their perceptions of the proposed changes in practice. Surveys were distributed digitally, with up to five email reminders sent to non-responders at 1- to 2-week intervals. Ten participants responded to the pre-study survey, and seven responded to the post-intervention survey.

Four months after the third workshop, all 12 participants were invited to online interviews: one group interview with four participants (50 minutes) and four individual interviews (8–20 minutes). Interview length varied depending on participants’ availability and the depth of their responses. Shorter interviews were concise and provided data relevant to the study aim. All interviews were conducted by J.N. H.L. and H.S. observed the first two interviews. Interviews followed a semi-structured interview guide (see Supplementary S2). All interviews were audio- and video-recorded using the communication platform's recording tool. The observer (J.N.) also collected workshop data through recordings and field notes documenting the workshop discussions and activities. The field notes were limited and captured key points and supported recall during analysis. Recordings allowed detailed review and were used as a complement to the field notes to support recall during analysis.

### Analysis

The interviews were transcribed verbatim and analysed using qualitative content analysis with an inductive approach [32]. Transcripts were repeatedly read and meaning units were identified, condensed and coded independently by each author, then discussed until consensus was reached through iterative comparison among the authors. The analysis was conducted by the researchers, with ongoing discussions and iterative refinement of codes and categories. We did not keep a formal audit trail. Codes from all five interviews were grouped into categories and subcategories agreed upon by all authors. An example of the coding process is provided in Supplementary material (see Supplementary S3).

Surveys included both quantitative and open-ended items. The quantitative items were analysed descriptively. Free text answers from the post-intervention survey were condensed and coded after interview analysis. Field notes and workshop recordings were used for the narrative description of the participatory process, providing examples of how principles such as co-creation and co-learning took shape. All data were pseudonymized, and participants were referred to as informants followed by a number and their profession in both interview and survey data. In the final stage of the analysis, the researchers brought together and compared findings from interviews, survey free-text responses, and field notes. Interview data constituted the primary data source, and survey data and field notes complemented and contextualized the interpretation of the categories. We did not perform member checking.

## Results

### Participatory health research process

Three online workshops were conducted with IP teams from two PHCCs, and three more with IP teams from three other PHCCs, each at 1-month intervals during the COVID-19 pandemic. In these workshops, the IP teams critically reviewed existing procedures and reflected on local improvements in osteoporosis management. The participatory health research process evolved over the course of the workshops. Discussions became increasingly participant-driven as they initiated questions and shared experiences. The exchange of ideas supported co-learning and co-creation and enabled teams to adapt work procedures to their local context. In later workshops, participants shared concrete results, such as a physician presenting a co-created osteoporosis management procedure ready for use and exchanging work procedures developed through earlier quality improvement efforts.

Analysis of interviews with eight participants resulted in three categories and seven subcategories (Table 2).

**Table 2:** Qualitative analysis: categories and subcategories identified in interviews.

Categories	Subcategories
**Innovative learning processes**	Creating work procedures adjusted to the local context Sharing experiences with staff from other PHCCs Perceiving increased competence and awareness of osteoporosis Increased interaction between staff members at the PHCC
**Perceived effects**	Patient benefit as perceived by participants Advanced role for nurses Thoughts on future improvements
**Factors influencing the task of changing work procedures**	

### Innovative learning processes

#### Creating work procedures adjusted to the local context

Participants described how local procedures were established specifying actions for each phase of investigation and treatment. Some existing procedures were retained because they worked well. They also clarified care processes by reviewing test panels, medication management, patient scheduling, and documentation in the electronic medical record system. Participants described adapting procedures to identify patients at risk of sustaining fragility fractures, including posting information on osteoporosis and fragility fractures on waiting room displays and actively checking hospital reports for relevant patients.

Erm… well, I guess we’ve become a bit more structured since taking part in this study, in terms of how we do things. We had a pretty good structure before, but now it's a bit better (Informant 3, nurse)

#### Sharing experiences with staff from other PHCCs

Several participants described taking part in the study with staff from other PHCCs as rewarding and providing insight into how others manage patients with osteoporosis. Participants reported that they shared problems, practical examples, and ideas on work procedures. One physician underlined the value of learning how others worked. Participants observed that the PHCCs were at different stages in terms of osteoporosis management; those who had recently implemented quality enhancements were keen to get more tips but appreciated confirmation that their own procedures were effective.

It was really helpful to hear some good ideas about how to go about things that might not be immediately obvious. Once you hear them, they’re self-evident, but they’re not things you’d necessarily have thought of yourself. (Informant 6, physician)

#### Perceiving increased competence and awareness of osteoporosis

Participants reported increased competence thanks to the work performed during the study and appreciated the initial online review. Both nurses and physicians stated that their own awareness and knowledge had increased and that the same was true of their colleagues. Several felt they had a better understanding of the complexity of the condition, including considering multiple parameters rather than just bone mineral density when deciding to start medication. Some physicians noted increased awareness of side effects and consulted outpatient osteoporosis clinics more often. Many of the participants found their work more satisfying, rewarding, comfortable, and secure. That said, greater knowledge was also a source of frustration, as participants felt that more could be done for this patient group given the resources to implement all measures, they considered necessary.

More knowledge. Erm, well, a better understanding (…) it's a complex issue with lots of different factors to consider, not just a DXA [Dual Energy X-ray Absorptiometry] value (…) it's improved my knowledge (…) (Informant 8, physician)

#### Increased interaction between staff members at the PHCC

Participants reported organizing IP team meetings to discuss changes to work procedures, including specific adjustments and documenting local routines. Collaboration with another professional group increased their understanding of each other's duties. One nurse noted an improvement in teamwork with staff not involved in the study and more frequent discussions on osteoporosis among nurses, leading to a more consistent approach. Some nurses said they had gained confidence. Participants also mentioned a stronger dialogue between physicians and nurses about patient cases and treatment. Some also trained colleagues and arranged educational sessions at their PHCCs.

Well, it's been a really good collaboration (…) nice to have the chance to work more closely with another category of staff and share experiences (…) and for me to, well, to learn… how things work at the PHCC from a nurse's perspective. (Informant 1, physician)

### Perceived effects

#### Patient benefit as perceived by participants

Participants reported identifying more patients at risk of fragility fractures, including during annual check-ups and care after discharge from hospital. Some patients sought care after seeing information on osteoporosis on waiting room displays. One physician observed that knowing medication doses could be adjusted helped ensure timely treatment. One nurse described that they were able to offer patients more convenient appointments as more nurses could provide treatment. Some nurses believed patients felt more confident about receiving timely care after a treatment scheduling system was introduced. Another physician mentioned that fewer blood samples were necessary once the procedure for ordering tests was optimized.

And then, I think we talk about it more now. Speaking just for myself, I’ve had quite a few patients with, for example, wrist fractures… who I’ve told: ‘No, I think we need to speak to a doctor about this. If no one else has done it, then we’ll do it - we’ll set up a phone call, and maybe you‘ll need Zoledronic acid as well’. (Informant 7, nurse)

#### Advanced role for nurses

Participants described initiating conversations at their PHCCs to clarify responsibility for patients at risk of fragility fractures. At one PHCC, one nurse already had a significant responsibility prior to the study. At others, according to most of the participants nurses took on greater responsibility over the course of the study, including greater autonomy to, for example, schedule appointments, order blood samples, and administer prescribed treatment. One physician noted that nurses managed patients with greater autonomy during treatment, only involving physicians when necessary or when treatment was complete. Another physician said that the osteoporosis clinic nurse had begun dealing with the initial appointment and examination, with the physician seeing patients only after all tests and assessments were complete. This physician added that the nurse had become an osteoporosis expert at the PHCC, sometimes more competent than physicians in managing these patients.

Erm … so, she [the nurse] now sees patients before they see the doctor, which is a bit of a new thing. She will have done all of the preparatory work which, hopefully, will make things much more straight-forward when the patient sees the doctor (…) by which time all the preparations have already been done, or however you like to put it. And she might even be able to decide that no further investigations are needed. (Informant 1, physician)

#### Thoughts on future improvements

Participants said they needed to keep working to identify more patients at risk of osteoporosis. Nurses at one PHCC saw opportunities to strengthen preventive work, including fall prevention. Nurses from two PHCCs wanted better information for patients and staff about, for example, drug side effects and activities at primary healthcare rehabilitation clinics. One physician highlighted the need for routine contact between PHCCs and hospital wards when patients are treated for fragility fractures. Physicians from two other PHCCs were considering evaluating osteoporosis management. One PHCC no longer had an osteoporosis nurse, but according to the physicians there were plans to recruit one.

Yes, well, we’ve got a good structure in place so we don’t lose track of any patients … that's fine… but spotting new patients, that's where we need to put in a bit more work. (Informant 3, nurse)

#### Factors influencing the task of changing work procedures

Participants mentioned several factors that could facilitate improvement. One nurse said that insights from the study itself could facilitate future improvements to procedures. Another noted that change was easier if everyone at the PHCC supported one another. Participants felt that a lack of staff and other resources hindered progress. Most participants reported finding it difficult to allocate working hours to change management. Participants at one PHCC felt that the diagnoses of other conditions had a higher priority; a nurse explained that as they already had an osteoporosis procedure, further development had not been prioritized.

But I think that insight into what we could do might lead to something positive, just knowing what we could change when we finally get the chance. (Informant 3, nurse)

Yes, exactly, we’re in a situation at our primary healthcare centre right now where we’ve had staff shortages (…) so it affects the work. (Informant 4, physician)

There are no subcategories in the final category. Few facilitating factors were mentioned; participants focused more on challenges. These findings were consistent with survey responses, where participants reported clearer work procedures and perceived positive effects for patients.

### Survey results

Free-text responses described similar changes in work procedures, including clearer and more structured assessment and treatment. One nurse wrote that many more patients were treated without a prior DXA than before the study. Overall, Likert scale ratings were equal or showed a slight increase post-intervention (see Supplementary S4). The most noticeable increase concerned whether the PHCC had clear and accessible work procedures for osteoporosis management. Among the items only included in post-intervention survey, one stood out: ‘The study has led to positive effects for patients at high risk of fragility fracture at my PHCC’. Most participants rated the impact as considerable or great. However, two nurses from one PHCC responded ‘to a small extent’, explaining in free-text that staff shortages and external factors were a barrier to putting the revised procedures into practice.

## Discussion

The main findings indicate that co-creation and collaboration within interprofessional teams enabled participants to revise and adapt osteoporosis management procedures locally. According to participants, these tailored procedures were perceived to improve patient care and contributed to the development of nursing roles. Participants also described increased knowledge and more collaborative care, not only in osteoporosis management but also in the management of other conditions. A social constructivist perspective views knowledge as co-created through interaction [28]. This perspective guided both the study design and analysis. The discussion addresses these findings in relation to participatory processes, learning, contextual factors, and interprofessional collaboration in primary healthcare.

Primary healthcare is complex, with evolving external requirements and local contexts [10]. Participatory health research can address this complexity by facilitating co-created, locally relevant knowledge through dialogue and reflection, empowering participants and supporting the continuous adaptation of work procedures to changing needs [27]. In participatory health research, problem-solving is often a key to knowledge creation [27]. Participants in this study faced two main challenges: identifying patients needing osteoporosis management and organizing care locally at their PHCC. Using a bottom-up approach, IP teams created procedures based on local needs and national guidelines in collaboration with other staff members. Teams engaged in ongoing dialogue, reflection, and adaptation during the participatory process. Some IP teams had introduced revised procedures by the end of the study, while others were preparing for local use. Participatory approaches are known to empower participants and support the local adaptation of healthcare processes, potentially facilitating continuous improvement and sustainability [8, 9, 27]. These findings reflect the levels of co-operation and co-learning described by Wright *et al*. [27]. Participants actively contribute to shaping both processes and outcomes within this framework. This study adds to previous participatory health research by showing how a facilitated workshop-based approach can support interprofessional teams in developing and adapting clinical work procedures within primary healthcare.

Both self-directed and participatory approaches promoted learning. The participatory approach enabled experiential learning, as participants actively co-created new procedures in workshops and through ongoing discussion at their PHCCs [13, 28]. The initial online review supported a theoretical understanding of osteoporosis management, which some participants found helpful. This supports the finding of Berger-Estelita *et al*. [12] that organizational support and social interaction enhance self-directed learning. Self-directed and experiential learning complemented one another, the former providing theoretical grounding and the latter helping to translate knowledge into practice. Participants described changes such as the adoption of co-created procedures and adjustments to clinical routines. These changes suggest experiences of higher-order learning; participants gained knowledge and shaped new working methods, which they then introduced into clinical practice [33].

Managerial support and expert facilitation are recognized as key enablers of successful change in clinical practice [34, 35]. In this study, operations managers were informed about the study's aims and the need for IP teams to allocate time to improving local osteoporosis management. A skilled moderator with expertise in osteoporosis management facilitated the workshop discussions. Despite this support, some IP teams struggled to prioritize the adaptation of procedures within their organization. Time constraints and heavy workloads were common; some IP teams worked through breaks or outside normal working hours, challenges previously reported as barriers to teamwork [30, 36]. The intervention relied on facilitated workshops and active participant engagement. This may influence its feasibility in routine clinical settings. The participatory approach allowed adaptation to local contexts. It may also require time, organizational support, and facilitation resources. These factors should be considered when transferring the approach to other settings.

Participants reported that their involvement clarified procedures and that some tasks were redistributed between physicians and nurses. They noted that this redistribution sometimes meant nurses taking on more advanced care delivery roles, which is consistent with previous findings from Swedish primary healthcare [3]. Physicians had overall medical responsibility and were contacted when necessary [3]. Many participants also noted increased interaction within IP teams and with other professionals in their PHCC. This suggests that applying ICP to care processes fosters interprofessional learning and strengthens relationships. This aligns with earlier research highlighting the importance of structured collaboration and clear role definitions when implementing interprofessional practices [7, 37]. Similar patterns have been reported in the management of other chronic conditions in primary healthcare. In these settings, team-based collaboration is well established [5, 7].

While participants expressed interest in improving osteoporosis management, some felt that not all colleagues shared their commitment. One participant noted that physicians were reluctant to assume additional responsibilities, reflecting concerns about clinical workload, a tendency observed previously [4].

### Strengths and limitations

To support credibility and confirmability, the analysis was conducted collaboratively. The researchers’ dual role in designing and facilitating the intervention, as well as conducting the analysis, may have introduced bias. The authors addressed this through continuous reflexive discussions. They moved back and forth between the data and their interpretations, discussing different understandings based on their varied professional backgrounds. Each researcher independently coded the data, followed by joint discussions to agree on categories and subcategories, repeatedly revisiting transcripts to ground interpretations in data [32]. Participants’ own words and expressions were used to stay close to intended meaning. Although participants were not involved in the analytical process, they actively shaped locally adapted work procedures, supporting participant validity by anchoring the work in practical knowledge and context [27]. Intersubjective validity was supported by the extent to which the research was perceived as credible and meaningful by the participants, particularly through their engagement in shaping the intervention [27]. Contextual validity was ensured by grounding the study in features of universal Swedish primary healthcare [27]. The reported improvements were based on participants’ perceptions rather than objective clinical outcomes. This limits conclusions about actual clinical impact on patient health and care quality. Participation was voluntary and may have attracted teams with a particular interest in osteoporosis care or interprofessional collaboration. This may have contributed to an overestimation of perceived positive effects. While this may increase relevance in the studied context, it may limit transferability. The study was conducted within Swedish primary healthcare, which may influence the transferability of the findings to other healthcare systems. The participatory approach and focus on interprofessional collaboration may be transferable to other primary healthcare settings and to the management of other chronic conditions. Local adaptation would be required. Transferability may be limited by contextual factors and the COVID-19 pandemic, which may have affected local uptake and the dependability of the results over time [32]. Catalytic validity was reflected in participants’ opportunities to influence and enact change in their own practice environments, and in the potential for new avenues of social action [27]. Ethical validity was supported by changes shaped by participants, ensuring outcomes were sound and just in local contexts [27].

While conducting workshops and interviews online enabled participation during the COVID-19 pandemic, it may have affected the quality of discussion and data richness [38]. This may have reduced opportunities for deeper interaction and observation compared to in-person formats. Although previous studies suggest that online interviews can compromise depth compared to in-person formats, this remains uncertain [38, 39]. Recruitment of PHCCs was challenging and time-consuming, probably due to the pandemic [40, 41]. The limited resources and time available to a doctoral project restricted the scope: only five PHCCs were recruited, and some interviews were short due to workload, potentially reducing data richness [38]. The number of participants was limited, which can be seen as a limitation in terms of information power [38]. The study aim was relatively broad, and an inductive approach was used, which may also have increased the need for more data [38].

The interview data were considered rich, and the later interviews did not add new aspects beyond those already identified in earlier interviews. All participants had taken part in the intervention and could describe their own experiences, which made the data highly relevant to the study aim and strengthened the information power [38]. The same findings were identified in both interviews and free-text responses. This strengthened the consistency of the results and aligned with recommendations for using multiple data sources in qualitative research [31].

The intervention required a skilled moderator to maintain engagement, which may limit scalability. Similar workshops would likely require coordination across multiple PHCCs or central support. The facilitated workshop-based approach could be applied in structured development initiatives, such as reviews of work procedures or quality improvement efforts, rather than as part of routine daily practice.

## Conclusion

The participatory approach enabled healthcare professionals to collaboratively improve osteoporosis management by leveraging local knowledge and fostering co-learning and interprofessional collaboration. According to our results, the process improved care delivery and role development among nurses and highlighted the importance of contextual adaptation and resource availability. Participatory health research can generate valuable insights for developing ICP to manage complex conditions such as osteoporosis.

## Supplementary Material

cmag045_Supplementary_Data

## Data Availability

Data underlying this article cannot be shared publicly due to ethical approval.

## Artificial intelligence-assisted technology

In writing this paper, the first author used Copilot to improve readability and provide text suggestions when needed. The authors have carefully reviewed, edited and revised the generated text, and take full responsibility for the content of the publication.
