# Supplementary material for: Co-creation and interprofessional collaborative practice for the local management of osteoporosis: a qualitative study in primary healthcare
Source: Fam Pract. 2026 Jun 23;43(4):cmag045. doi: 10.1093/fampra/cmag045 (PMC13287522; doi:10.1093/fampra/cmag045)
Supplement: cmag045_Supplementary_Data [file cmag045_supplementary_data.pdf]

Supplementary S1A: Pre-intervention Survey

1. Your gender is (Female/Male/Other)
2. Your year of birth is (Drop-down with year)
3. What is your profession? (Physician/Nurse)

4. A fall is defined as an event in which someone unintentionally ends up on the floor or ground, regardless of whether or not it results in injury.
Do you have a nurse at your workplace who works extensively with patients who may need fall-prevention measures? (Yes/No)

5. A fragility fracture is defined as a fracture of the vertebra, hip, pelvis, upper arm, or wrist occurring in a person aged 50 or older as the result of low energy trauma from, for example, a fall on a flat surface.
Do you have a nurse at your workplace who works extensively with patients who may need measures to prevent fragility fractures? Yes/No

Choose the option that you think best describes each statement (not at all / to a small extent / to a considerable extent / to a great extent / don’t know):

- 6a. At my primary healthcare centre, we are good at identifying people who may need measures to prevent fragility fractures.
- 6b. At my primary healthcare centre, we are good at offering measures to prevent fragility fractures.
- 6c. At my primary healthcare centre, nurses and doctors work together extensively with people who may need measures to prevent fragility fractures.
- 6d. At my primary healthcare centre, we have clear routines accessible to everyone regarding how people who may need measures to prevent fragility fractures should be cared for and who is responsible for each part of the care.

Supplementary S1B: Post-intervention Survey

1. Your gender is (Female/Male/Other)
2. Your year of birth is (Drop-down with year)
3. What is your profession? (Physician/Nurse)

4. A fall is defined as an event in which someone unintentionally ends up on the floor or ground, regardless of whether or not it results in injury.
Do you have a nurse at your workplace who works extensively with patients who may need fall-prevention measures? (Yes/No)

5. A fragility fracture is defined as a fracture of the vertebra, hip, pelvis, upper arm, or wrist occurring in a person aged 50 or older as the result of low energy trauma from, for example, a fall on a flat surface.
Do you have a nurse at your workplace who works extensively with patients who may need measures to prevent fragility fractures? Yes/No

Choose the option that you think best describes each statement (not at all / to a small extent / to a considerable extent / to a great extent / don't know):

- 6a. At my primary healthcare centre, we are good at identifying people who may need measures to prevent fragility fractures.
- 6b. At my primary healthcare centre, we are good at offering measures to prevent fragility fractures.
- 6c. At my primary healthcare centre, nurses and doctors work together extensively with people who may need measures to prevent fragility fractures.
- 6d. At my primary healthcare centre, we have clear routines accessible to everyone regarding how people who may need measures to prevent fragility fractures should be cared for and who is responsible for each part of the care.
- 7. To what extent do you feel that your proposed changes have been implemented in practice?
- 8. The project has had positive effects for **those working at my primary healthcare centre**.
- 9. The project has had positive effects for **me in my work at the primary healthcare centre**.
- 10. The project has had positive effects at **my primary healthcare centre for patients who may need measures to prevent fragility fractures.**

11. Can you give specific examples of changes at the primary healthcare centre that explain your answer to question 10? (Free text)

12. Can you give examples of things that worked well in your own or your team’s work during this project? (Free text)

13. Can you give examples of things you would have liked to do differently if you had the opportunity to start the project over? (Free text)

Supplementary S2: Interview Guide for Group and Individual Interviews.

1. What made you decide to get involved in this project? (go round the group)
2. Can you describe how you have organised work with patients at high risk of fracture at your workplace? Please discuss among yourselves.
3. How do you perceive that the care of patients at high risk of fracture has changed since you started working on your procedures as part of this research project?
   Probing question: Can you give examples of factors that have facilitated or hindered change?
4. How do you experience collaboration with the physician/nurse since you started working on your work procedures in this research project?
   Probing question: Can you give examples of how you have collaborated with the physician/nurse between the workshops?
5. How do you currently view the boundaries of your responsibilities as a physician or a nurse in work with patients with high fracture risk?
6. What is your experience of working with patients at high risk of fracture today?
7. Is there anything else you would like to bring up?

Supplementary S3: Example of Coding Tree

Example of the qualitative coding process

Category	Subcategories	Codes
Innovative learning process	Perceiving increased competence and awareness of osteoporosis	As a doctor I notice increased awareness among nurses
	Creating work processes adjusted to the local context	We have created more structure and a clearer workflow
	Sharing experiences with staff from other PHCCs	Staff from other PHCCs gave new, useful ideas
	Increased interaction between staff members at the PHCC	I [physician] talk more with the nurse about what we should do according to the work process

PHCC = Primary Healthcare Centre

DXA = Bone mineral density scan

Supplementary S4: Results from surveys before and after intervention

Frequency of nurse specialised on fall and fracture prevention

**Items from the surveys before and after intervention**	**Yes, before (n=10)**	**Yes, after (n=7)**
Do you have a nurse at your PHCC who works extensively with patients who may need **fall-prevention** measures?	10 %	29 %
Do you have a nurse at your PHCC who works extensively with patients who may need measures to prevent **fragility fractures**?	30 %	57 %

PHCC = Primary healthcare centre

Structured osteoporosis management at the PHCCs

Items from the surveys before (N=10) and after (N=7) the intervention

Item 6a. At my PHCC, we are good at identifying people who may need measures to prevent fragility fractures

Item 6b. At my PHCC, we are good at offering measures to prevent fragility fractures.

Item 6c. At my PHCC, nurses and doctors work together extensively with people who may need measures to prevent fragility fractures.

Item 6d. At my PHCC, we have clear routines accessible to everyone regarding how people who may need measures to prevent fragility fractures should be cared for and who is responsible for each part of the care.

Results: sum of median scores for items 6a – 6d, before 10 points and after 13,5 points

PHCC = Primary healthcare centre

Participants' perceived effects of the intervention

**Items from survey after intervention**	**After (n=7): Median (Range)**
To what extent do you perceive your proposed changes have been implemented in practice?	3 (2-4)
The project has had positive effects for **those working at my PHCC**.	3 (2-4)
The project has had positive effects for **me in my work at the PHCC**.	3 (2-4)
The project has had positive effects at **my PHCC for patients who may need measures to prevent fragility fractures.**	4 (2-4)

Likert scale: 1 = Not at all, 2 = To a small extent, 3 = To a considerable extent, 4 = To a great extent.
PHCC = Primary healthcare centre

The SRQR reporting checklist

For checking that qualitative health research articles can be understood and used by everyone

> **Note**
>
> If you have not used a reporting guideline before, read about how and why to use them and check whether SRQR is the most applicable reporting guideline for your work.
>
> Reporting guidelines are most useful when used early in research. When writing a manuscript or application, consider using the Full Guidance where you'll see explanations and examples for each item.
>
> After writing, demonstrate adherence by completing this checklist:
>
> 1. Specify where each item is described (see Note 1).
> 2. Cite this checklist (See Note 2).
> 3. Include your completed checklist as a supplement when submitting to a journal so that future readers can use it to find information.

	Item Description	Page/Location (or reason for not reporting)
**Title & Abstract**		
Title	Describe the nature and topic of the study. Identify the study as qualitative or indicate the approach or data collection methods.	Title page
Abstract	Summarise the key elements of the study using the abstract format of the intended publication.	Abstract
**Introduction**		
Problem Formulation	Describe the problem/phenomenon studied, its significance, relevant theory and empirical work, and gaps in current knowledge.	Background
Purpose or research question	Describe the purpose of the study and specific objectives or questions.	Background
**Methods**		
Qualitative approach and	Describe your qualitative approach, your guiding theory (if appropriate), and	Methods – Design section

research paradigm	research paradigm, and reasons for your choices.	Methods – Analysis section<br>Discussion
Researcher characteristics and reflexivity	Describe how researchers' characteristics may influence the research, including personal attributes, qualifications/experience, relationship with participants, assumptions, and/or presuppositions; potential or actual interaction between researchers' characteristics and the research questions, approach, methods, results and/or transferability.	Methods – Researcher Characteristics and Reflexivity section<br>Methods – Analysis section
Context	Describe the setting/site(s) in which the study was conducted, why it was selected, and any other salient contextual factors that may influence the study.	Methods – Context section
Sampling strategy	Describe how and why research participants, documents, or events were selected; criteria for deciding when no further sampling was necessary, and the rationale for those criteria.	Methods – Participants section<br>Discussion – Strengths and limitations section
Ethical issues pertaining to human subjects	Describe any approval by an appropriate ethics review board and participant consent, or explain any lack thereof. Describe any other confidentiality and data security issues.	Declarations – Ethical Approval section
Data collection methods	Describe the types of data collected; details of data collection procedures including (as appropriate) start and stop dates of data collection and analysis, iterative process, **triangulation** of sources/methods, and modification of procedures in response to evolving study findings. Describe your rationale for these choices.	Methods – Intervention section<br>Methods – Data collection section<br>Methods – Analysis section<br>Discussion – Strengths and limitations section
Data collection instruments and technologies	Describe any instruments (e.g., interview guides, questionnaires) and devices (e.g., audio recorders) used for data collection; describe if/how the instrument(s) changed over the course of the study.	Methods – Data Collection section<br>Supplementary material S1 Supplementary material S2
Units of study	Describe the number and relevant characteristics of participants, documents, or events included in the study. Describe the level of participation.	Methods – Context section<br>Methods – Participants section

		Methods – Intervention section<br>Table 1
Data processing	Describe the methods for processing data prior to and during analysis, including transcription, data entry, data management and security, verification of data integrity, data coding, and anonymisation / deidentification of excerpts.	Methods – Analysis section<br>Methods – Data collection section<br>Declarations – Ethical approval section
Data analysis	Describe the process by which inferences, themes, etc. were identified and developed, including the researchers involved in data analysis; usually references a specific paradigm or approach. Describe why you chose this process.	Methods – Analysis section<br>Discussion – Strengths and limitations section
Techniques to enhance trustworthiness	Describe any techniques to enhance trustworthiness and credibility of data analysis,(e.g., member checking, triangulation, audit trail). Describe why you chose these techniques.	Methods – Data collection section<br>Methods – Analysis section<br>Discussion – Strengths and limitations section
**Results**		
Synthesis and interpretation	Describe the main findings (e.g., interpretations, inferences, and themes); might include development of a theory or model, or integration with prior research or theory.	Results<br>Table 2<br>Supplementary material S4
Links to empirical data	Provide evidence (e.g., quotes, field notes, text excerpts, photographs) to substantiate analytic findings.	Results<br>Table 2<br>Supplementary material S4
**Discussion**		
Integration with prior work, implications, transferability, and contribution(s) to the field	Summarize the main findings, explain how findings and conclusions connect to, support, elaborate on, or challenge conclusions of earlier scholarship; discuss the scope of application/generalizability; identify unique contribution(s) to scholarship in a discipline or field.	Discussion<br>Conclusion
Limitations	Discuss the trustworthiness and limitations of findings	Discussion – Strengths and limitations
**Other**		

Conflicts of interest	Describe any potential sources of influence or perceived influence on study conduct and conclusions. Describe how these were managed.	Declaration – Conflict of Interest section
Funding	Describe sources of funding and other support. Describe the role of funders in data collection, interpretation, and reporting.	Declaration – Funding section

1 How to specify where content is

Tell the reader where they can find information. E.g.,

- Results; paragraph 2
- Methods, Participants; paragraphs 1 & 2.
- Table 3
- Supplement B, para. 4

If you have chosen not to describe an item, explain why. You can do this in the checklist, or as a note below it.

You can describe items in the article body, or in tables, figures, or supplementary materials, and should prioritize items you feel are most important to your intended audience. The order of items in your manuscript does not need to match the order of items in this checklist. You can decide how best to structure your work.

2 How to cite

Describe how you used SRQR at the end of your Methods section, referencing the resources you used e.g.,

> ‘We used the SRQR reporting guideline(1) to draft this manuscript, and the SRQR reporting checklist(2) when editing, included in supplement A’

If you use a reporting checklist, remember to include it as a supplement when publishing so that readers can easily find information and see how you have interpreted the guidance.

1. O’Brien BC, Harris IB, Beckman TJ, Reed DA, Cook DA. Standards for reporting qualitative research: A synthesis of recommendations. Academic Medicine [Internet]. 2014 Sep;89(9):1245–51. Available from: https://journals.lww.com/academicmedicine/fulltext/2014/09000/Standards_for_Reporting_Qualitative_Research__A.21.aspx

2. O’Brien BC, Harris IB, Beckman TJ, Reed DA, Cook DA. The SRQR reporting checklist. In: Harwood J, Albury C, Beyer J de, Schlüssel M, Collins G, editors. The EQUATOR network reporting guideline platform [Internet]. The UK EQUATOR Centre; 2025. Available from: https:/resources.equator-network.org/reporting-guidelines/srqr/srqr-checklist.docx
